# Upregulation of FOXM1 in a subset of relapsed myeloma results in poor outcome

**DOI:** 10.1038/s41408-018-0060-0

**Published:** 2018-02-15

**Authors:** Chunyan Gu, Carol Holman, Ramakrishna Sompallae, Xuefang Jing, Michael Tomasson, Dirk Hose, Anja Seckinger, Fenghuang Zhan, Guido Tricot, Hartmut Goldschmidt, Ye Yang, Siegfried Janz

**Affiliations:** 1 The Third Affiliated Hospital, Nanjing University of Chinese Medicine, 210023 Nanjing, China; 2 Department of Pathology, The University of Iowa Roy J. and Lucille A. Carver College of Medicine, Iowa City, 52242 Iowa USA; 3 Iowa Institute for Genetics, The University of Iowa Roy J. and Lucille A. Carver College of Medicine, Iowa City, 52242 Iowa USA; 4 Department of Internal Medicine, The University of Iowa Roy J. and Lucille A. Carver College of Medicine, Iowa City, 52242 Iowa USA; 5 Holden Comprehensive Cancer Center, The University of Iowa Roy J. and Lucille A. Carver College of Medicine, Iowa City, 52242 Iowa USA; 6 Medizinische Klinik V, Universitätsklinikum Heidelberg, 69120 Heidelberg, Germany; 7 Nationales Centrum für Tumorerkrankungen, 69120 Heidelberg, Germany; 8 Key Laboratory of Acupuncture and Medicine Research, Ministry of Education, Nanjing University of Chinese Medicine, Nanjing, 210023 China

Following up on our previous work demonstrating the involvement of the transcription factor, forkhead box M1 (FOXM1), in the biology and outcome of a high-risk subset of newly diagnosed multiple myeloma (nMM)^1^, we sought to determine whether upregulation of *FOXM1* may also be a feature of relapsed myeloma (rMM). To that end, we analyzed the total therapy 2 (TT2) dataset (GSE2658) from the University of Arkansas for Medical Science, which contains microarray-based gene expression and clinical outcome data on 88 patients with known *FOXM1* mRNA levels at baseline and relapse. Statistical comparison demonstrated a significant increase (*p* = 10^−4^) in mean (by ~70%) and median (~2.9-fold) *FOXM1* expression upon relapse (Fig. 1a, left). Just like in nMM^1^, the distinction between high and low *FOXM1* message at relapse was of prognostic value: *FOXM1*^High^ patients (*n* = 11, 12.5%) exhibited markedly reduced event-free survival and overall survival compared to *FOXM1*^Low^ patients (*n* = 77, Fig. 1a, right). To confirm these results with the help of an independent dataset that is more recent than TT2, we took advantage of myeloma patients from the University of Heidelberg, Germany, which were uniformly treated upfront using HDT/ASCT and new myeloma drugs. In this cohort of 692 patients with nMM, *FOXM1*^High^ status was as negative a predictor of survival (Fig. 1b) as in the original study^1^. Furthermore, relapsed myelomas from the Heidelberg cohort (*n* = 55) also demonstrated significant elevations of *FOXM1* message compared to nMM (Fig. 1c, left) and *FOXM1*^High^ status at relapse—found in 8 of 60 (13%) patients—led to an even more dramatic reduction in survival than seen in the TT2 cohort (Fig. 1c, right). These findings led us to conclude that upregulation of *FOXM1* is a molecular marker of a subset of relapsed myelomas that results in inferior outcome in patients with peak expression levels.

**Fig. 1:**
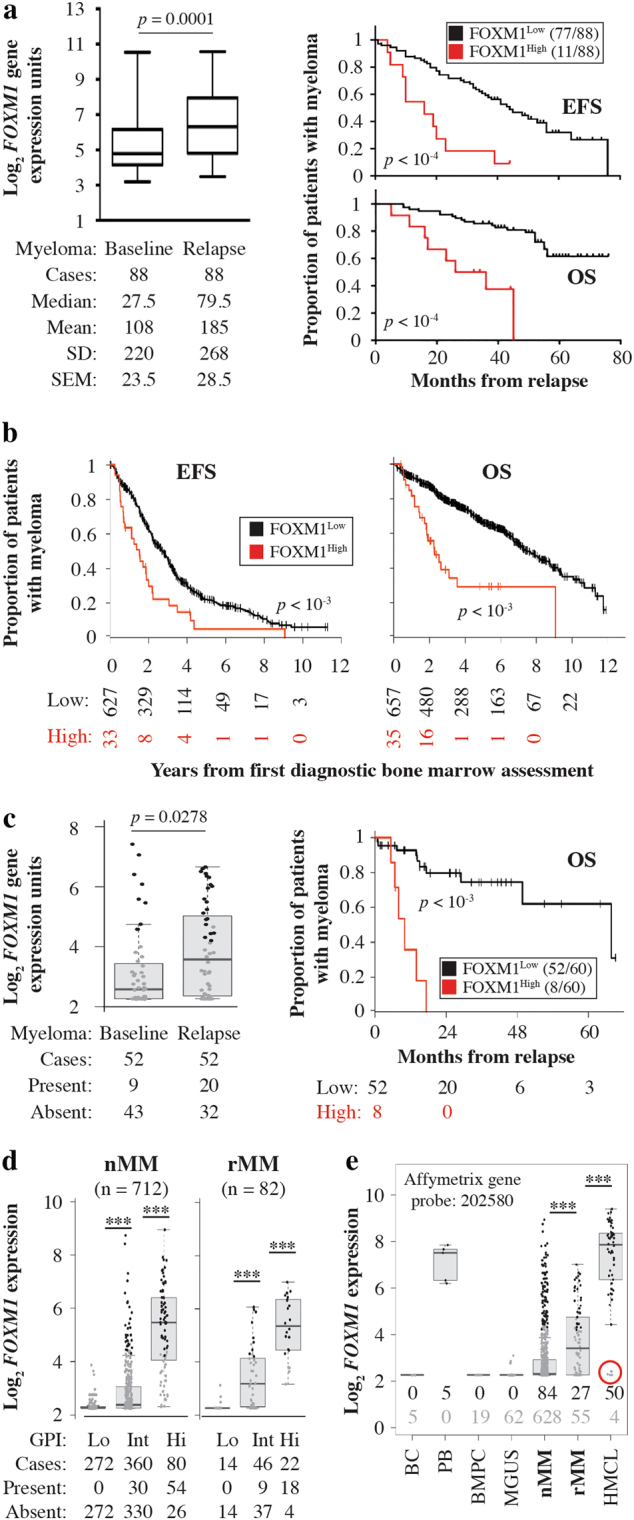
Expression of *FOXM1*, a proliferation gene in normal and neoplastic plasma cells, is elevated in relapsed myeloma and predicts poor outcome in the subset of patients with peak expression levels. **a** Shown on the left is a box plot of *FOXM1* mRNA levels determined by microarray analysis (gene probe set 202580_x_at) of magnetic bead-sorted CD138^+^ myeloma cells collected from 88 patients of the UAMS Total Therapy 2 (TT2) trial at baseline (nMM) and relapse (rMM). Median *FOXM1* expression is indicated by a black horizontal line within the box. The 25th and 75th percentiles of gene expression are indicated by lower and upper horizontal lines, respectively. Mean *FOXM1* expression, standard deviation of the mean (SD) and standard error of the mean (SEM) are indicated below the plot. The increase in *FOXM1* message was driven by 61 of 88 (69%) patients; in 20 patients *FOXM1* mRNA was down at relapse compared to baseline and in 7 patients there was no change. Hence, upregulation of *FOXM1* at relapse is a feature of the majority of cases, but it is not a universal phenomenon. Presented on the right is a Kaplan-Meier analysis of event-free survival (EFS) and overall survival (OS) of rMM patients stratified into cases containing *FOXM1* message above the median (red curve, FOXM1^High^) or below the median (black curve, FOXM1^Low^). **b** High *FOXM1* expression prognosticates poor survival of patients with nMM in the University of Heidelberg cohort. Myeloma patients were assigned to 2 groups distinguished by low *FOXM1* mRNA levels (FOXM1^Low^) and high *FOXM1* mRNA levels (FOXM1^High^) based on microarray measurements. The decline in EFS (left) and OS (right) after the first diagnostic bone marrow assessment is plotted. Survival was determined using Cox’s proportional hazard model. EFS and OS are significantly reduced in FOXM1^High^ patients compared to FOXM1^Low^ patients (*p* < 10^−3^). **c** *FOXM1* expression in 52 paired nMM vs. rMM samples from the University of Heidelberg cohort is depicted to the left. Just like in the TT2 sample presented in (**a**), *FOXM1* at relapse was up in the majority of patients (35/56, 63%), but down in 17 (30%) patients and unchanged in 4 patients. Shown to the right is a plot of OS, determined using Cox’s proportional hazard model, of patients with FOXM1^High^ relapse (*n* = 8) and FOXM1^Low^ relapse (*n* = 52). Maximally selected rank statistics was used to stratify patients with rMM using 6 as cutoff. The proportion of FOXM1^High^ relapse (13.3%) is similar to that in panel a (12.5%). **d** Box plots comparing *FOXM1* mRNA levels in CD138^+^ fractionated myeloma cells obtained from 712 patients with newly diagnosed disease (nMM) and 82 patients with relapsed disease (rMM) from the Heidelberg cohort. Based on the GPI score, patients were stratified into 3 distinct groups of low, intermediate and high proliferation. *FOXM1* expression was different in these groups at *p* < 10^−3^ (indicated by 3 asterisks). The proportion of cases in which *FOXM1* was present (expressed) or absent (not expressed), based on a cutoff determined by PANP, is indicated for all three groups below the plots. While *FOXM1* was not present in the low proliferation group of both new and relapsed disease, it was more frequently present in rMM compared to nMM the intermediate (9/46 or 20% vs. 30/360 or 8%) and high (18/22 or 82% vs. 54/80 or 68%) proliferation groups. **e** Box plot depicting median *FOXM1* expression in B cells (BC, *n* = 5), plasmablasts (PB, *n* = 5) and bone marrow plasma cells (BMPC, *n* = 19) from healthy individuals, or in CD138^+^ BMPCs from patients with monoclonal gammopathy of undetermined significance (MGUS, *n* = 62), newly diagnosed myeloma (nMM, *n* = 712) and relapsed myeloma (rMM, *n* = 54) from the Heidelberg cohort. A panel of human myeloma cell lines (HMCL, *n* = 54), which represent the terminal disease stage of plasma cell leukemia, is included for comparison. Presence and absence of *FOXM1* is indicated for all groups using black and grey numbers, respectively. The circumstance that in 4 of 54 (7%) HMCLs *FOXM1* was absent (indicated by red circle) suggests that malignant plasma cells may switch to a FOXM1-independent proliferation program, perhaps following secondary genetic change during serial in vitro propagation of cells

Because (1) FOXM1 is best known as positive regulator of cell cycle progression^2^, (2) tumor cell proliferation is an important, independent, adverse determinant of myeloma patient survival^3^ and (3) myeloma relapse from chemotherapy ultimately results in selection of cell clones with increased proliferation rates^4^, we decided to evaluate whether increased *FOXM1* expression at relapse may be associated with increased myeloma proliferation. For this purpose, we relied on a validated gene expression-based proliferation index (GPI) of myeloma^3^ to stratify 712 patients with nMM and 82 patients with rMM from the Heidelberg cohort into three different groups defined by low, intermediate or high proliferation rates of tumor cells. Fig. 1d shows that *FOXM1* expression paralleled the GPI score in both nMM (left panel) and rMM (right panel). High *FOXM1* message levels, which even surpassed those measured in GPI^High^ myeloma, were also observed in normal plasmablasts derived from cytokine-induced peripheral blood B-lymphocytes of healthy donors and in rapidly dividing HMCLs (human myeloma cell lines) propagated in vitro (Fig. 1e). These results were consistent with the contention that FOXM1 regulates cell cycle progression in both normal and neoplastic plasma cells.

Recurrent cancer including relapsed myeloma is frequently associated with therapy resistance acquired by changes in genetic pathways wherein FOXM1 has been repeatedly implicated^2^. With this in mind, we asked whether upregulation of FOXM1 may decrease the sensitivity of myeloma cells to the widely used myeloma drugs, bortezomib (Bz) and doxorubicin (Dox). We chose two HMCLs, XG1 and CAG – engineered to overexpress (OE) lentivirus-encoded FOXM1 (FOXM1^OE^ myeloma) or containing normal (N) levels of the transcription factor due to transduction with “empty” virus (FOXM1^N^ myeloma)—as experimental model system. We found that FOXM1^OE^ cells exhibited partial resistance to both drugs. Specifically, the half-maximal inhibitory concentration (IC_50_) of Bz was 5.6-fold (CAG) and 1.9-fold (XG1) higher in FOXM1^OE^ than FOXM1^N^ cells, whereas the corresponding difference upon treatment with Dox was 2.9-fold (CAG) and 1.5-fold (XG1). Flow cytometric measurements of annexin V, a marker of apoptosis, were in agreement with these results, as FOXM1^OE^ cells invariably experienced less drug-induced death than FOXM1^N^ cells. Additionally, FOXM1^OE^ cells demonstrated enhanced clonogenic growth in soft agar and increased ABC-transporter drug efflux-pump activity by eFluxx-ID Gold MDR analysis. These results—not shown here but to be presented in greater depth in a future publication—indicated that FOXM1 promotes drug resistance in myeloma. Our findings further suggested that suppression of FOXM1 may re-sensitize myeloma to killing by genotoxic drugs, as seen in other types of cancer^2^.

Taking into account that (1) the mechanism by which FOXM1 promotes drug resistance in myeloma may involve increased proliferation of myeloma cells, (2) our previous work implicated the G1-to-S cell cycle regulator, cyclin-dependent kinase 6 (CDK6), in the genetic network of FOXM1 in myeloma^1^, (3) new findings indicate that *CDK6* and *FOXM1* are co-expressed in myeloma and, when used as 2-gene mini-signature, predictive of patient survival (Supplemental Fig. 1) and, finally, (4) CDK6 is a key upstream activator of the retinoblastoma Rb/E2F (E2F transcription factor 1) axis, a key regulator of the cell cycle and pathways of senecscence and drug resistance—we decided to determine whether FOXM1 interacts physically with Rb in myeloma. Co-IP analysis showed that antibody to Rb (bait) pulled down FOXM1 in myeloma cells (Fig. 2a, top), whereas antibody to FOXM1 (bait) pulled down Rb (Fig. 2a, bottom). Western analysis of paired FOXM1^OE^ and FOXM1^N^ samples demonstrated that total and phosphorylated Rb were increased in FOXM1^OE^ cells, with higher levels seen in XG1 than CAG cells (Fig. 2b, top). Conversely, Rb was lower in myeloma in which FOXM1 had been “knocked down” (FOXM1^KD^) by RNA interference (Fig. 2b, bottom). These results supported the contention that FOXM1 regulates, in part, the Rb/E2F axis in myeloma. Bearing in mind that FOXM1-targeted inhibitors suitable for clinical application are not available at this juncture, the results further suggested that pharmacological targeting CDK6 may afford an indirect, alternative approach to inhibiting FOXM1^High^ myeloma.

**Fig. 2:**
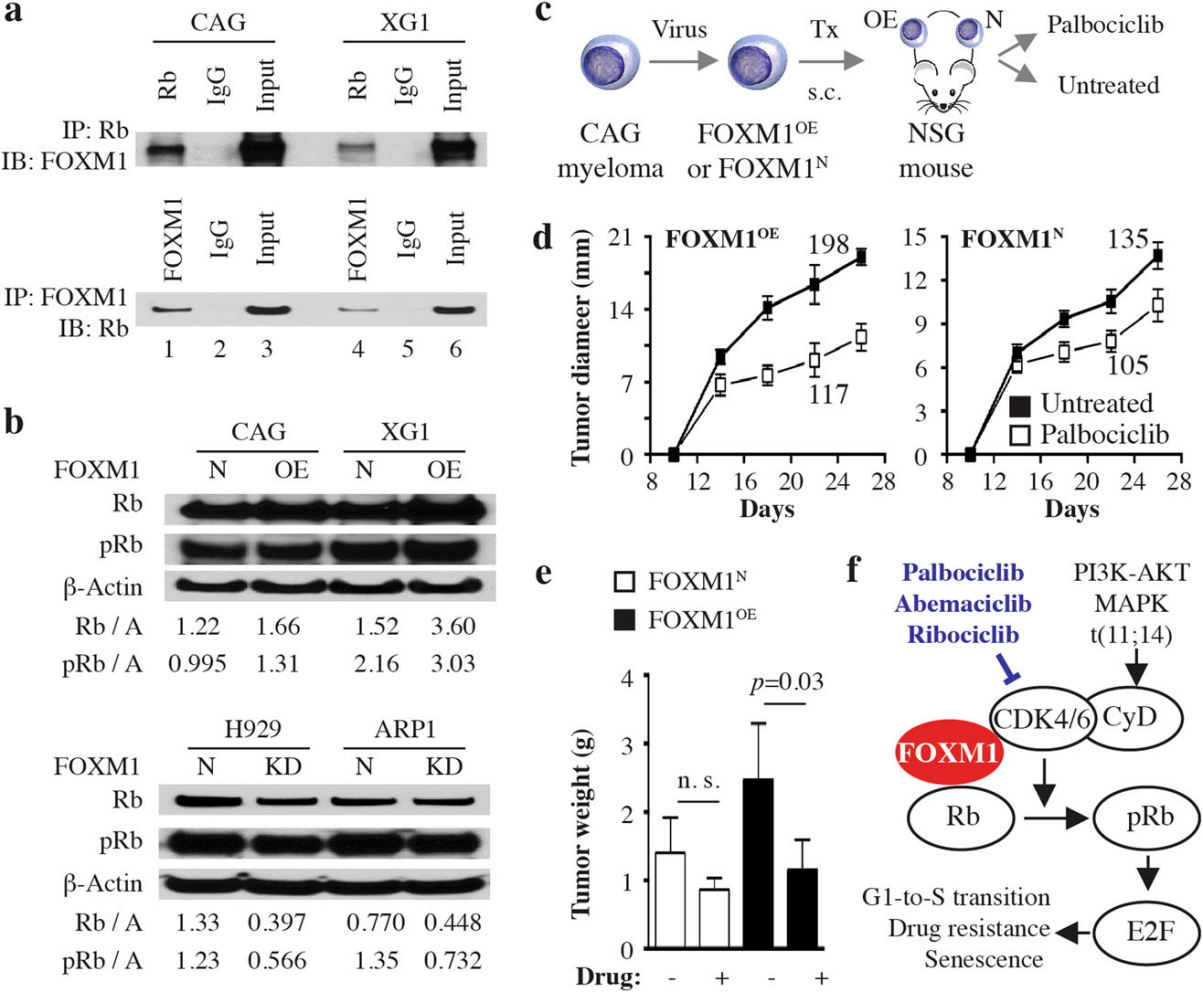
FOXM1 interacts with the cyclin D-CDK4/6-Rb-E2F pathway in myeloma. **a** FOXM1 binds Rb (official gene symbol, *RB1*) in myeloma cells. Co-immunoprecipitation (Co-IP) result indicating physical interaction of Rb and FOXM1 in CAG and XG1 myeloma cells. Immunoblots using a specific IgG antibody (Ab) to FOXM1 (following IP with Ab to Rb) or Rb (following IP with Ab to FOXM1) are shown on top of each other. An IgG isotype control is labeled “IgG.” Whole cell lysates not subjected to Co-IP (Input) were included as additional control. **b** Shown on top is a Western blot of total Rb and its activated, phosphorylated form (pRb) in FOXM1^OE^ and FOXM1^N^ CAG and XG1 myeloma cells. A similar blot containing samples of FOXM1^KD^ and FOXM1^N^ H929 and ARP1 myeloma cells is presented at bottom. The abundance of Rb and pRb, relative to β-actin, was determined using densitometry. The ratios are indicated below the blots. **c** Study scheme for assessing the efficacy with which the small-drug CDK4/6 inhibitor, palbociclib, inhibits human myeloma growth in immunodeficient mice. FOXM1^OE^ and FOXM1^N^ CAG cells were xenografted s.c. into the right and left flank of NSG mice (*n* = 10), respectively. Beginning on day 7 after myeloma cell transfer, five mice were treated with daily i.p. injections of the CDK4/6 inhibitor, PD0332991, until study end on day 26. Five mice were left untreated. Tumor diameters were measured on days 14, 18, 22, and 26 after xenografting, using a caliper. **d** Time course of tumor growth in hosts. Mean tumor diameters (squares) and standard deviations of the mean (short vertical lines with error bars) are plotted. The area under the curve (AUC), ranging from 105 to 198, is indicated for all study groups. **e** Mean tumor weight at study end. FOXM1^OE^ xenografts treated with PD (1.14 ± 0.456 g) were significantly smaller than their untreated counterparts (2.46 ± 0.833 g) using Mann–Whitney’s *t* test (*p* = 0.032). FOXM1^N^ xenografts also showed a difference (0.862 ± 0.172 g vs. 1.40 ± 0.515 g) but missed the 5% threshold of statistical significance (*p* = 0.095). Median tumor weights in the four groups were significantly different by Kruskall-Wallis analysis (*p* = 0.0178). **f** Working model on the interaction of FOXM1 with CDK4/6 and Rb in myeloma. FOXM1 is a proliferation-associated transcription factor that interacts with the cyclin D-CDK4/6-Rb-E2F pathway, a key regulator of the G1-to-S cell cycle transition. Upstream signals activating this pathway include those emanating from MAPK-ERK and PI3K-AKT-mTOR signaling and, in a subset of myeloma, deregulated cyclin D expression following from chromosomal translocation. In addition to cell cycle progression, the Rb/E2F-dependent transcriptional program regulates cellular senescence and response to drug treatment. Small-molecule CDK4/6 inhibitors, such as palbocicblib, abemacicblib and ribocicblib, that have the targeting of the ATP-binding pocket of CDK4 and CDK6 in common, may lend themselves to new treatment approaches to FOXM1^High^ myeloma. Palbocicblib and the CDK5-targeted inhibitor dinacicblib (not shown) are currently undergoing clinical testing in myeloma

To follow up on that, we determined the efficacy with which the selective, potent, orally administered, small-molecule inhibitor of CDK6 and CDK4^5^, palbociclib (PD0332991), hampers myeloma growth in vivo. Since an accurate mouse model of relapsed human myeloma is not available at this juncture, we assessed palbociclib with the assistance of a surrogate model, HMCL-in-mouse xenografting (Fig. 2c). Although the drug hindered the growth of both FOXM1^OE^ and FOXM1^N^ xenografts (Fig. 2d), the former were more sensitive than the latter. Using the area under the growth curve as metric of drug efficacy, palbociclib inhibited FOXM1^OE^ tumors by ~41% (117 vs. 198) and FOXM1^N^ tumors by ~22% (105 vs. 135). Similarly, at study endpoint, drug-dependent growth inhibition of FOXM1^OE^ xenografts (1.14 ± 0.456 g mean tumor weight) amounted to 54% (relative to untreated controls: 2.46 ± 0.833 g), whereas FOXM1^N^ xenografts were inhibited by 38% (0.862 ± 0.172 g vs. 1.40 ± 0.515 g; Fig. 2e). These results provided preclinical support for the potential benefit of a CDK4/6-targeted therapy for FOXM1^High^ myeloma. This is in line with studies by other investigators who demonstrated that palbociclib arrested cell cycle progression of patient-derived tumor cells at G1 in vitro^6^ and, in combination with Bz, inhibited myeloma-like 5T33 mouse tumors in vivo^7^. Fig. 2f depicts a working model on the interaction of FOXM1 with the CDK4/6-Rb-E2F pathway in myeloma. Included are 3 small-molecule inhibitors of CDK4/6 that are in clinical use or advanced stages of clinical testing^8^.

The results presented in Fig. 2 are in line with encouraging evidence on the efficacy of CDK4/6 inhibitors in solid cancers^9^ and the ongoing clinical assessment of these drugs in blood cancers including MM. In regards to myeloma, the outcome of the recently completed phase 1/2 clinical trial of palbociclib in rMM (NCT00555906)^8^—and interim results of new trials that are currently recruiting (NCT02030483, NCT02693535)—are eagerly awaited. Of great additional import is the finding of the Mayo Phase 2 Consortium that dinaciclib, a potent inhibitor of CDK5 involved in acquired resistance to proteasome inhibition in myeloma^10^, exhibits single-agent activity in rMM^11^. Because dinaciclib blocks cell division at later stages of the mitotic cycle (late G1, S, and G2) than palbociclib does (early G1), therapeutic synergism of these inhibitors is possible. To better put the promise of CDK-targeted treatments for FOXM1^High^ myeloma into perspective, it is helpful to recognize that FOXM1 is critically involved in the natural history and outcome of other B-cell neoplasms, such as acute lymphoblastic leukemia^12^, diffuse large cell lymphoma^13^ and follicular lymphoma^14^. The broad significance of FOXM1 for B-lineage malignancies agrees with its pleiotropic function in cancer biology, impacting—in addition to cell cycle progression—DNA damage repair, self-renewal of stem cell-like cancer cells, and response to both cytostatic and targeted treatments^2^. Finally, FOXM1 is a crucial determinant of cancer outcome; e.g., a recent pan-cancer meta-analysis of ~18,000 gene expression signatures identified the FOXM1 regulatory network as a major predictor of adverse outcomes across 39 solid and hematologic malignancies, including MM^15^.

## Electronic supplementary material

Supplemental Figure 1
