# Supplementary figures and images for: Upregulation of FOXM1 in a subset of relapsed myeloma results in poor outcome
Source: Blood Cancer J. 2018 Feb 15;8(2):22. doi: 10.1038/s41408-018-0060-0 (PMC5814454; doi:10.1038/s41408-018-0060-0)

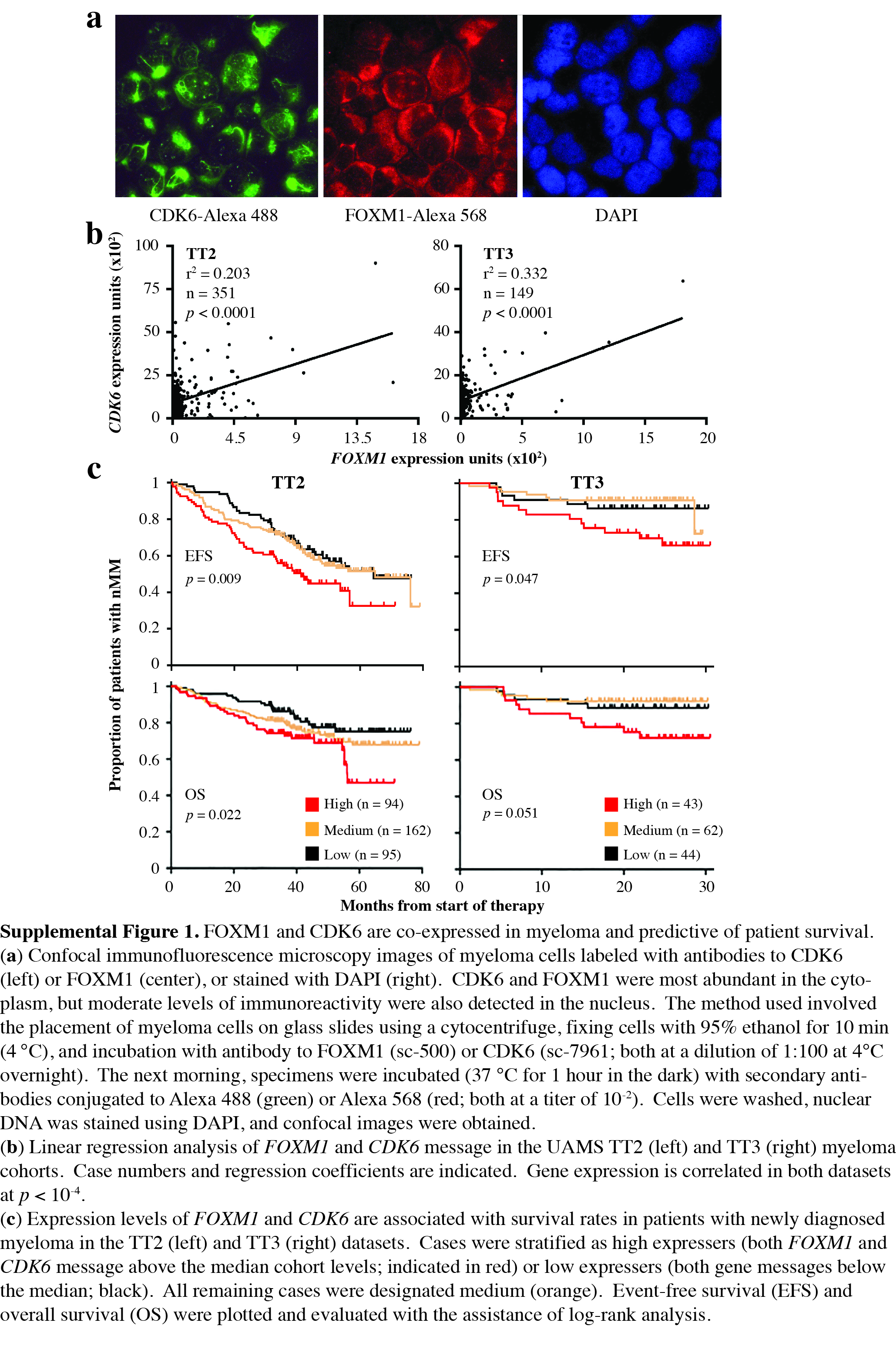

Supplement: Supplementary file 1 — Supplemental Figure 1 [file 41408_2018_60_MOESM1_ESM.jpg]
